# Fragranced consumer products: effects on autistic adults in the United States, Australia, and United Kingdom

**DOI:** 10.1007/s11869-018-0625-x

**Published:** 2018-09-25

**Authors:** Anne Steinemann

**Affiliations:** 1 Department of Infrastructure Engineering, Melbourne School of Engineering, The University of Melbourne, Parkville, Victoria 3010 Australia; 2 College of Science and Engineering, James Cook University, Townsville, Queensland 4811 Australia; 3 Climate, Atmospheric Sciences, and Physical Oceanography, Scripps Institution of Oceanography, University of California, San Diego, La Jolla, CA 92093 USA

**Keywords:** Autism, Autism spectrum disorder, ASD, Autistic, Fragranced consumer products, Indoor air quality, Fragrance, Health effects, Volatile organic compounds

## Abstract

Fragranced consumer products, such as cleaning supplies, air fresheners, and personal care products, can have adverse effects on both air quality and health. This study investigates the effects of fragranced products on autistic individuals ages 18–65 in the United States, Australia, and United Kingdom. Nationally representative population surveys (*n* = 1137; 1098; 1100) found that, across the three countries, 4.3% of adults (*n* = 142) report medically diagnosed autism (2.3%), an autism spectrum disorder (2.4%), or both. Of these autistic adults, 83.7% report adverse health effects from fragranced products, including migraine headaches (42.9%), neurological problems (34.3%), respiratory problems (44.7%), and asthma attacks (35.9%). In particular, 62.9% of autistic adults report health problems from air fresheners or deodorizers, 57.5% from the scent of laundry products coming from a dryer vent, 65.9% from being in a room cleaned with scented products, and 60.5% from being near someone wearing a fragranced product. Health problems can be severe, with 74.1% of these effects considered potentially disabling under legislation in each country. Further, 59.4% of autistic adults have lost workdays or lost a job, in the past year, due to fragranced product exposure in the workplace. More than twice as many autistic as well as non-autistic individuals would prefer that workplaces, health care facilities, and health care professionals were fragrance-free rather than fragranced. Results show that vulnerable individuals, such as those with autism or autism spectrum disorders, can be profoundly, adversely, and disproportionately affected by exposure to fragranced consumer products.

**Electronic supplementary material:**

The online version of this article (10.1007/s11869-018-0625-x) contains supplementary material, which is available to authorized users.

## Introduction

Fragranced consumer products are ubiquitous in society and emit numerous volatile organic compounds including hazardous air pollutants (Steinemann 2015, Nazaroff and Weschler 2004). Exposure to fragranced consumer products (or fragranced products, for brevity) has been associated with a range of adverse health effects in the general population and in vulnerable sub-populations. Nationally representative studies of the general population in the United States (US), Australia (AU), and United Kingdom (UK) found that 34.7%, 33.0%, and 27.8%, respectively, of adults report one or more types of health problems when exposed to fragranced products such as air fresheners, laundry supplies, cleaning products, personal care products, and household items (Steinemann 2016, 2017, 2018a). These adverse health effects include migraine headaches, asthma attacks, neurological problems, and respiratory difficulties, among others. Further, asthmatics are especially vulnerable, with 64.3% and 55.6% of asthmatics in the US and AU, respectively, reporting adverse health effects from fragranced products (Steinemann 2018b, Steinemann et al. 2018).

Little prior research has investigated links between autism and fragranced products. Bagasra et al. (2013) found that fragrance chemicals can induce neurotoxic and neurostimulatory effects, even at exceptionally low concentrations. Sealey et al. (2015) demonstrated that fragrance compounds can cause neuromodifications, as well as depletion of OXY and AVP receptor-positive neurons in male but not female neuroblastoma cell lines, consistent with a male bias in autism. Heilbrun et al. (2015) showed that chemically intolerant, including fragrance sensitive, mothers were three times more likely to have a child with autism.

This study investigates the effects of exposure to fragranced products on adults diagnosed with autism or autism spectrum disorder (ASD) or both, across three countries (US, AU, UK). In addition to the prevalence, types, and severity of health effects, the study also investigates the loss of access in society and loss of workdays and jobs, due to exposure to fragranced consumer products. Results from this study reveal an important and underexplored connection between fragranced products and autism and ASD. Results also accentuate a relatively straightforward way to reduce adverse health, economic, and societal effects for autistic individuals, as well as non-autistic individuals, by reducing use and exposure to common fragranced consumer products.

## Methods

In three separate national studies, an online survey was conducted with sample populations (*n* = 1137 US; 1098 AU; 1100 UK) statistically representative of the general population according to age, gender, and region (confidence limit = 95%, confidence interval = 3% for all studies). Using randomized participant recruitment of adults ages 18–65, the surveys drew upon large web-based panels held by Survey Sampling International (SSI 2018), with over 5,000,000 US, 200,000 AU, and 950,000 UK people. The survey instrument was developed and tested over a 2-year period before full implementation in June 2016. The survey response rate was 94% US, 93% AU, 97% UK, and all responses were anonymous. The research study received ethics approval from the University of Melbourne. Details on the survey methodology are provided as a supplemental document.

To determine the sub-population of autistic individuals, the survey asked, “Has a doctor or health care professional ever told you that you have autism or autism spectrum disorder?” The survey further asked whether the diagnosis was for autism, ASD, or both. In this article, the term “autistic adults” (or “autistic individuals”) will refer to those medically diagnosed with autism, an autism spectrum disorder, or both. This term was selected to represent the pooled sub-populations (of autism and/or ASD) and alleviate potential confusion with individual sub-populations. The term “non-autistic adults” (or “autistic individuals”) will be those in the general population other than autistic adults; that is, individuals who do not report a medical diagnosis of autism or ASD, or who report “don’t know/not sure” or “decline to answer” in the survey. The term “fragrance sensitivity” will refer to adverse health effects from exposure to fragranced consumer products.

It is recognized that diagnostic criteria and terminology regarding autism and ASD vary considerably by location and by time. The intent of this study is not to investigate or define those diagnoses and terms, but rather to identify those individuals reporting a medical diagnosis of autism and/or ASD and then examine the co-morbidity with fragrance sensitivity.

Survey questions investigated exposures to fragranced products, associated health effects, effects of exposure to fragranced products in the workplace and in society, preferences for fragrance-free environments and policies, and demographic information.

Specific exposure contexts included air fresheners or deodorizers used in public restrooms and other environments, the scent of laundry products coming from a dryer vent, being in a room after it was cleaned with scented cleaning products, being near someone wearing a fragranced product, entering a business with the scent of fragranced products, fragranced soap used in public restrooms, and ability to access environments that used fragranced products.

Fragranced products were categorized as follows: (a) air fresheners and deodorizers (e.g., sprays, solids, oils, disks); (b) personal care products (e.g., soaps, hand sanitizer, lotions, deodorant, sunscreen, shampoos); (c) cleaning supplies (e.g., all-purpose cleaners, disinfectants, dishwashing soap); (d) laundry products (e.g., detergents, fabric softeners, dryer sheets); (e) household products (e.g., scented candles, restroom paper, trash bags, baby products); (f) fragrance (e.g., perfume, cologne, after-shave); and (g) other.

Health effects were categorized as follows: (a) migraine headaches; (b) asthma attacks; (c) neurological problems (e.g., dizziness, seizures, head pain, fainting, loss of coordination); (d) respiratory problems (e.g., difficulty breathing, coughing, shortness of breath); (e) skin problems (e.g., rashes, hives, red skin, tingling skin, dermatitis); (f) cognitive problems (e.g., difficulties thinking, concentrating, or remembering); (g) mucosal symptoms (e.g., watery or red eyes, nasal congestion, sneezing); (h) immune system problems (e.g., swollen lymph glands, fever, fatigue); (i) gastrointestinal problems (e.g., nausea, bloating, cramping, diarrhea); (j) cardiovascular problems (e.g., fast or irregular heartbeat, jitteriness, chest discomfort); (k) musculoskeletal problems (e.g., muscle or joint pain, cramps, weakness); and (j) other.

Descriptive statistics and cross tabulations determined the prevalence of outcomes according to different countries and sub-populations. Chi-squared analyses compared proportions among the three countries to determine whether a statistically significant difference exists. Prevalence odds ratios (PORs) measured the strength of the association between reported health effects among autistic and non-autistic individuals to determine whether one group is proportionally more affected. All chi-squared analyses and prevalence odds ratios were calculated using a 95% confidence interval.

## Results

Main findings are presented in this section, along with summary tables. Complete data and statistical analyses for each country individually and across the three countries are provided as supplemental documentation.

### Study populations and prevalence

Of the general population surveyed, 4.3% (*n* = 142) report being medically diagnosed with autism (2.3%), an autism spectrum disorder (2.4%), or both. Of these autistic adults, 83.7% report adverse health effects from fragranced consumer products. (See Table 1.)

**Table 1:** Study populations: autistic individuals in the United States (US), Australia (AU), and United Kingdom (UK)

	US	AU	UK	Total
Total (*n*) general population	1137	1098	1100	3335
Total (*n*, %) sub-populations				
Autistic (autism/ASD)	49	41	52	142
	4.3%	3.7%	4.7%	4.3%
Autism	25	24	27	76
	2.2%	2.2%	2.5%	2.3%
Autism spectrum disorder (ASD)	26	26	29	81
	2.3%	2.4%	2.6%	2.4%
Non-autistic	1088	1057	1048	3193
	95.7%	96.3%	95.3%	95.7%
Not with autism/ASD	1073	1025	1021	3119
	94.4%	93.4%	92.8%	93.5%
Don’t know/not sure	13	30	26	69
	1.1%	2.7%	2.4%	2.1%
Decline to answer	2	2	1	5
	0.2%	0.2%	0.1%	0.1%

Across the three countries, no statistically significant difference was found in terms of the prevalence of autism and ASD (*p* > 0.05, chi-square test).

### Health problems from fragranced consumer products

Among autistic adults, 83.7% across the three countries (83.7% US, 82.9% AU, 84.6% UK) report one or more types of adverse health effects from exposure to one or more types of fragranced products. The most common adverse health effects were respiratory problems (44.7%); migraine headaches (42.9%); mucosal symptoms (42.1%); skin problems (39.7%); asthma attacks (35.9%); cardiovascular problems (34.3%); and neurological problems (34.3%). (See Table 2.) Across the three countries, no statistically significant difference was found in the prevalence of fragrance sensitivity among autistic adults (*p* > 0.05, chi-square test). Among non-autistic adults, 29.5% report one or more types of adverse health effects from exposure to one or more types of fragranced products. Among all types of health effects, autistic adults are more likely to be affected than non-autistic adults (prevalence odds ratio [POR] 12.05; 95% confidence interval [CI] 7.66–18.95).

**Table 2:** Types of health problems from exposure to fragranced consumer products for autistic and non-autistic individuals

	Autistic individuals	Non-autistic individuals
Total (*n*) autistic/non-autistic individuals	142	3193
Health problems from fragranced products (*n*, %)	119	943
Fragrance sensitivity	83.7%	29.5%
Autistic individuals: US (*n* = 41, 83.7%); AU (*n* = 34, 82.9%); UK (*n* = 44, 84.6%) Non-autistic individuals: US (*n* = 353, 32.4%); AU (*n* = 328, 31.0%); UK (*n* = 262, 25%)		
Types of health problems from exposure to fragranced consumer products		
* Migraine headaches	42.9%	10.0%
* Asthma attacks	35.9%	6.2%
* Neurological problems (e.g., dizziness, seizures, head pain, fainting, loss of coordination)	34.3%	3.8%
* Respiratory problems (e.g., difficulty breathing, coughing, shortness of breath)	44.7%	14.3%
* Skin problems (e.g., rashes, hives, red skin, tingling skin, dermatitis)	39.7%	8.7%
* Cognitive problems (e.g., difficulties thinking, concentrating, or remembering)	32.5%	3.0%
* Mucosal symptoms (e.g., watery or red eyes, nasal congestion, sneezing)	42.1%	11.9%
* Immune system problems (e.g., swollen lymph glands, fever, fatigue)	31.4%	1.8%
* Gastrointestinal problems (e.g., nausea, bloating, cramping, diarrhea)	29.2%	2.8%
* Cardiovascular problems (e.g., fast or irregular heartbeat, jitteriness, chest discomfort)	34.3%	2.1%
* Musculoskeletal problems (e.g., muscle or joint pain, cramps, weakness)	34.1%	1.4%
* Other	2.0%	1.9%
Health problems from fragranced consumer products are potentially disabling	74.1%	25.4%
Autistic individuals: US (n = 35, 85.4%); AU (*n* = 28, 82.4%); UK (*n* = 24, 54.5%) Non-autistic individuals: US (*n* = 160, 45.3%); AU (n = 34, 10.4%); UK (*n* = 54, 20.6%)		

Severity of health effects from exposure to fragranced products was investigated using the criteria for disability according to each country’s disability legislation (ADAAA 2008; DDA 1992; EA 2010). Among autistic adults reporting health effects, 74.1% across the three countries (85.4% US, 82.4% AU, 54.5% UK) report that the severity of these health effects from fragranced products was potentially disabling. Among non-autistic adults, the prevalence of potentially disabling effects was 25.4%. (See Table 2.) Thus, autistic adults were more likely to report disabling health effects from fragranced products than non-autistic adults (POR 7.62; 95% CI, 4.95–11.72).

### Problematic exposures, societal access, and workplace effects

Specific problematic exposures, associated with adverse health effects for autistic adults, include but are not limited to the following: air fresheners and deodorizers (62.9%), the scent of laundry products coming from a dryer vent (57.5%), being in a room recently cleaned with scented products (65.9%), being near someone wearing a fragranced product (60.5%), and other types of fragranced consumer products (64.3%). (See Table 3.)

**Table 3:** Health problems, societal access, and workplace effects from exposure to fragranced consumer products for autistic and non-autistic individuals

	Autistic individuals	Non-autistic individuals
Total (*n*) autistic/non-autistic individuals	142	3193
Health problems from fragranced products (*n*, %)	119	943
	83.7%	29.5%
Health problems from exposure to		
Air fresheners or deodorizers	62.9%	15.4%
Scent of laundry products from a dryer vent	57.5%	37.0%
Room cleaned with scented products	65.9%	45.0%
Someone wearing a fragranced product	60.5%	17.0%
Any other type of fragranced consumer product	64.3%	16.8%
Societal access and workplace effects		
Unable to use restrooms in public place because of air freshener or scented product	62.1%	11.6%
Unable to wash hands because of fragranced soap	59.8%	9.4%
Leave a business quickly because of fragranced product	58.7%	14.8%
Prevented from going someplace because of fragranced product	66.7%	14.9%
Lost workdays or a job in past year due to fragranced product exposure in workplace	59.4%	7.6%
Supportive of fragrance-free policy in the workplace	65.5%	46.1%
Prefer fragrance-free health care facilities and professionals	77.2%	45.8%

Fragranced consumer products were associated with lost access in society and lost workdays and jobs: 62.1% of autistic adults are unable or reluctant to use the restrooms in a public place if it has an air freshener, deodorizer, or scented product; 59.8% are unable or reluctant to wash their hands with soap in a public place if the soap is fragranced; 58.7% enter a business and then want to leave as quickly as possible if they smell air fresheners or a fragranced product; and 66.7% have been prevented from going someplace because they would be exposed to a fragranced product that would make them sick. (See Table 3.)

Importantly, 59.4% of autistic adults have lost workdays or a job, in the past year, due to illness from fragranced product exposure in the workplace. Fragrance-free policies receive a strong majority of support among both autistic and non-autistic adults. Among autistic adults, 65.5% would support a fragrance-free policy in the workplace (compared to 24.0% that would not). Also, 77.2% of autistic adults would prefer that health care facilities and health care professionals be fragrance-free (compared to 16.4% that would not). Among non-autistic adults, 46.1% would support a fragrance-free workplace (compared with 21.6% that would not). Also, 45.8% of non-autistic adults would prefer that health care facilities and health care professionals be fragrance-free (compared to 25.1% that would not).

Of the 83.7% of autistic adults reporting adverse health effects from fragranced products, proportionately more males (64.6%) report adverse effects than females (35.4%), relative to the general population (48.6% males, 51.4% females). (See supplementary tables.)

Study strengths include the following: (a) sample populations in each country were statistically representative of age, gender, and region; (b) respondents were randomly recruited from large web-based panels developed from multiple sources to reflect population characteristics; (c) the survey employed questions from large national studies in the US, AU, and UK previously conducted and published; and (d) the study populations presented statistically similar proportions of autistic individuals and autistic individuals with fragrance sensitivity, across the three countries.

Study limitations include the following: (a) only adults (ages 18–65) were included in the survey, which excludes children and the elderly; (b) the survey relied on self-reported data; however, self-report is a widely accepted and standard method for survey research; (c) the cross-sectional design of the survey limits the ability to follow responses over time without additional surveys, (d) all possible fragranced products and health effects were not included, although the relatively low percentages for responses in the “other” categories indicate the survey captured the primary products and effects.

## Discussion

Fragranced consumer products have been associated with adverse health and societal effects in the general population and especially in vulnerable populations. Results from three countries indicate that autistic individuals are profoundly, adversely, and disproportionately affected by exposure to fragranced consumer products. While non-autistic adults are also affected, autistic adults are more likely to report adverse and disabling health effects from exposure.

Involuntary exposure is a concern. Autistic individuals are prevented from accessing public restrooms, societal venues, businesses, and workplaces due to adverse health effects from fragranced products. Further, 59.4% of autistic adults have lost workdays or a job, in the past year, due to fragranced product exposure in the workplace. A strong majority of autistic as well as non-autistic adults would prefer that workplaces, health care facilities, and health care professionals were fragrance-free than fragranced.

Results of this study add to the growing evidence that fragranced consumer products can harm health for both autistic and non-autistic individuals, with autistic individuals more affected. Further research is needed to understand why these types of products are apparently inducing a range of adverse effects. In the meantime, to reduce or prevent negative health, societal, and economic effects, a sensible and effective approach would be to limit exposure to fragranced consumer products.

## Electronic supplementary material

ESM 1 (PDF 158 kb)

ESM 2 (PDF 52 kb)
