# Supplementary material for: Fragranced consumer products: effects on autistic adults in the United States, Australia, and United Kingdom
Source: Air Qual Atmos Health. 2018 Sep 25;11(10):1137–42. doi: 10.1007/s11869-018-0625-x (PMC6244938; doi:10.1007/s11869-018-0625-x)
Supplement: Supplementary file 1 — (PDF 158 kb) [file 11869_2018_625_MOESM1_ESM.pdf]

Table 1	Autistic			Non-Autistic			Autistic	Non-Autistic
Country							US, AU, UK	US, AU, UK
	US	AU	UK	US	AU	UK		
Total	49	41	52	1088	1057	1048	142	3193
	4.300%	3.700%	4.700%	95.700%	96.300%	95.300%	4.3%	95.7%
US	1137	-	-	1137	-	-		
	100.00%	-	-	100.00%	-	-		
AU	-	1098	-	-	1098	-		
	-	100.00%	-	-	100.00%	-		
UK	-	-	1100	-	-	1100		
	-	-	100.00%	-	-	100.00%		

Table 2	Autistic			Non-Autistic			Autistic US, AU, UK	Non-Autistic US, AU, UK
What is your gender?	US	AU	UK	US	AU	UK		
Total	49	41	52	1088	1057	1048		
	100.00%	100.00%	100.00%	100.00%	100.00%	100.00%		
Male	33	24	34	492	519	516		
	67.30%	58.50%	65.40%	45.20%	49.10%	49.20%	63.7%	47.8%
Female	16	17	18	596	538	532		
	32.70%	41.50%	34.60%	54.80%	50.90%	50.80%	36.3%	52.2%

Table 3	Autistic			Non-Autistic			Autistic US, AU, UK	Non-Autistic US, AU, UK
What is your age?	US	AU	UK	US	AU	UK		
Total	49	41	52	1088	1057	1048		
	100.00%	100.00%	100.00%	100.00%	100.00%	100.00%		
18-24 (21)	9	7	10	116	149	154		
	18.40%	17.10%	19.20%	10.70%	14.10%	14.70%	18.2%	13.2%
25-34 (29.5)	16	14	25	249	225	206		
	32.70%	34.10%	48.10%	22.90%	21.30%	19.70%	38.3%	21.3%
35-44 (39.5)	20	10	11	271	246	231		
	40.80%	24.40%	21.20%	24.90%	23.30%	22.00%	28.8%	23.4%
45-54 (49.5)	3	9	5	249	232	238		
	6.10%	22.00%	9.60%	22.90%	21.90%	22.70%	12.6%	22.5%
55-65 (60)	1	1	1	203	205	219		
	2.00%	2.40%	1.90%	18.70%	19.40%	20.90%	2.1%	19.7%

Table 4

US Region.

	US			US		
**Total**	49			1088		
	100.00%			100.00%		
**Northeast**	9			198		
	18.40%			18.20%		
**Midwest**	11			235		
	22.40%			21.60%		
**South**	18			404		
	36.70%			37.10%		
**West**	11			251		
	22.40%			23.10%		

Table 5

AUTRALIA Region.

	AU	AU
**Total**	41	1057
	100.00%	100.00%
**Australian Capital Territory**	-	20
	-	1.90%
**New South Wales**	15	345
	36.60%	32.60%
**Northern Territory**	-	7
	-	0.70%
**Queensland**	9	208
	22.00%	19.70%
**South Australia**	2	83
	4.90%	7.90%
**Tasmania**	1	24
	2.40%	2.30%
**Victoria**	8	270
	19.50%	25.50%
**Western Australia**	6	100
	14.60%	9.50%

Table 6

UK Region.

			UK			UK
**Total**			52			1048
			100.00%			100.00%
**East Anglia**			4			95
			7.70%			9.10%
**East Midlands**			5			72
			9.60%			6.90%
**London**			10			144
			19.20%			13.70%
**North East**			3			41
			5.80%			3.90%
**Northern Ireland**			2			31
			3.80%			3.00%
**North West**			7			114
			13.50%			10.90%
**Scotland**			2			97
			3.80%			9.30%
**South East**			6			137
			11.50%			13.10%
**South West**			3			85
			5.80%			8.10%
**Wales**			2			53
			3.80%			5.10%
**West Midlands**			6			93
			11.50%			8.90%
**Yorkshire & Humberside**			2			86
			3.80%			8.20%

Table 7	Autistic			Non-Autistic			Autistic US, AU, UK	Non-Autistic US, AU, UK
Q2. Do you experience any health problems when exposed to air fresheners or deodorizers?								
	US	AU	UK	US	AU	UK		
Total	49	41	52	1088	1057	1048		
	100.00%	100.00%	100.00%	100.00%	100.00%	100.00%		
Yes	33	23	34	199	157	136		
	67.30%	56.10%	65.40%	18.30%	14.90%	13.00%	62.9%	15.4%
No	11	13	11	780	747	787		
	22.40%	31.70%	21.20%	71.70%	70.70%	75.10%	25.1%	72.5%
Don't know/not sure	4	5	7	105	152	123		
	8.20%	12.20%	13.50%	9.70%	14.40%	11.70%	11.3%	11.9%
Decline to answer	1	-	-	4	1	2		
	2.00%	-	-	0.40%	0.10%	0.20%	2.0%	0.2%

Table 8	Autistic			Non-Autistic			Autistic US, AU, UK	Non-Autistic US, AU, UK
	US	AU	UK	US	AU	UK		
Total	33	23	34	199	157	136		
	100.00%	100.00%	100.00%	100.00%	100.00%	100.00%		
Migraine headaches	14	9	10	68	37	30		
	42.40%	39.10%	29.40%	34.20%	23.60%	22.10%	37.0%	26.6%
Asthma attacks	6	12	9	47	37	35		
	18.20%	52.20%	26.50%	23.60%	23.60%	25.70%	32.3%	24.3%
Neurological problems (e.g., dizziness, seizures,	11	5	7	25	19	8		
head pain, fainting, loss of coordination)	33.30%	21.70%	20.60%	12.60%	12.10%	5.90%	25.2%	10.2%
Respiratory problems (e.g., difficulty breathing,	11	10	7	97	90	69		
coughing, shortness of breath)	33.30%	43.50%	20.60%	48.70%	57.30%	50.70%	32.5%	52.2%
Skin problems (e.g., rashes, hives, red skin,	11	8	11	54	45	39		
tingling skin, dermatitis)	33.30%	34.80%	32.40%	27.10%	28.70%	28.70%	33.5%	28.2%
Cognitive problems (e.g., difficulties thinking,	10	7	7	21	14	7		
concentrating, or remembering)	30.30%	30.40%	20.60%	10.60%	8.90%	5.10%	27.1%	8.2%
Mucosal symptoms (e.g., watery or red eyes, nasal	11	10	7	75	58	42		
congestion, sneezing)	33.30%	43.50%	20.60%	37.70%	36.90%	30.90%	32.5%	35.2%
Immune system problems (e.g., swollen lymph	11	9	4	10	11	6		
glands, fever, fatigue)	33.30%	39.10%	11.80%	5.00%	7.00%	4.40%	28.1%	5.5%
Gastrointestinal problems (e.g., nausea, bloating,	10	6	10	21	10	7		
cramping, diarrhea)	30.30%	26.10%	29.40%	10.60%	6.40%	5.10%	28.6%	7.4%
Cardiovascular problems (e.g., fast or irregular	12	11	14	18	10	9		
heartbeat, jitteriness, chest discomfort)	36.40%	47.80%	41.20%	9.00%	6.40%	6.60%	41.8%	7.3%
Musculoskeletal problems (e.g., muscle or joint	14	7	8	13	11	3		
pain, cramps, weakness)	42.40%	30.40%	23.50%	6.50%	7.00%	2.20%	32.1%	5.2%
Other	1	-	1	7	6	6		
	3.00%	-	2.90%	3.50%	3.80%	4.40%	3.0%	3.9%

BA. Which of the following health problems do you experience?

Base: Respondents who experienced below health problems when exposed to air fresheners or deodorizers

Table 9

Q3. Do you experience any health problems from the scent of laundry products coming from a dryer vent?

	Autistic			Non-Autistic			Autistic US, AU, UK	Non-Autistic US, AU, UK
	US	AU	UK	US	AU	UK		
**Total**	49	41	52	1088	1057	1048		
	100.00%	100.00%	100.00%	100.00%	100.00%	100.00%		
**Yes**	35	21	26	107	46	40		
	71.40%	51.20%	50.00%	9.80%	4.40%	3.80%	57.5%	37.0%
**No**	14	18	20	892	891	906		
	28.60%	43.90%	38.50%	82.00%	84.30%	86.50%	37.0%	84.3%
**Don't know/not sure**	-	2	6	88	118	101		
	-	4.90%	11.50%	8.10%	11.20%	9.60%	8.2%	9.6%
**Decline to answer**	-	-	-	1	2	1		
	-	-	-	0.10%	0.20%	0.10%		

Table 10

BA. Which of the following health problems do you experience?

Base: Respondents who experienced below health problems from the scent of laundry products coming from a dryer vent

	Autistic			Non-Autistic			Autistic US, AU, UK	Non-Autistic US, AU, UK
	US	AU	UK	US	AU	UK		
Total	35	21	26	107	46	40		
	100.00%	100.00%	100.00%	100.00%	100.00%	100.00%		
Migraine headaches	9	5	7	28	9	8		
	25.70%	23.80%	26.90%	26.20%	19.60%	20.00%	25.5%	21.9%
Asthma attacks	7	8	9	21	7	14		
	20.00%	38.10%	34.60%	19.60%	15.20%	35.00%	30.9%	23.3%
Neurological problems (e.g., dizziness, seizures,	10	5	5	14	3	3		
head pain, fainting, loss of coordination)	28.60%	23.80%	19.20%	13.10%	6.50%	7.50%	23.9%	9.0%
Respiratory problems (e.g., difficulty breathing,	12	9	7	34	19	14		
coughing, shortness of breath)	34.30%	42.90%	26.90%	31.80%	41.30%	35.00%	34.7%	36.0%
Skin problems (e.g., rashes, hives, red skin,	14	9	6	27	14	11		
tingling skin, dermatitis)	40.00%	42.90%	23.10%	25.20%	30.40%	27.50%	35.3%	27.7%
Cognitive problems (e.g., difficulties thinking,	7	7	9	8	4	2		
concentrating, or remembering)	20.00%	33.30%	34.60%	7.50%	8.70%	5.00%	29.3%	7.1%
Mucosal symptoms (e.g., watery or red eyes, nasal	11	6	7	37	11	5		
congestion, sneezing)	31.40%	28.60%	26.90%	34.60%	23.90%	12.50%	29.0%	23.7%
Immune system problems (e.g., swollen lymph	13	11	5	6	9	4		
glands, fever, fatigue)	37.10%	52.40%	19.20%	5.60%	19.60%	10.00%	36.2%	11.7%
Gastrointestinal problems (e.g., nausea, bloating,	13	5	7	16	8	-		
cramping, diarrhea)	37.10%	23.80%	26.90%	15.00%	17.40%	-	29.3%	16.2%
Cardiovascular problems (e.g., fast or irregular	6	9	4	9	6	2		
heartbeat, jitteriness, chest discomfort)	17.10%	42.90%	15.40%	8.40%	13.00%	5.00%	25.1%	8.8%
Musculoskeletal problems (e.g., muscle or joint	16	8	6	7	2	2		
pain, cramps, weakness)	45.70%	38.10%	23.10%	6.50%	4.30%	5.00%	35.6%	5.3%
Other	1	-	-	3	2	1		
	2.90%	-	-	2.80%	4.30%	2.50%	2.9%	3.2%

Table 11	Autistic			Non-Autistic			Autistic US, AU, UK	Non-Autistic US, AU, UK
Q4. Do you experience any health problems from being in a room after it has been cleaned with scented products?								
	US	AU	UK	US	AU	UK		
Total	49	41	52	1088	1057	1048		
	100.00%	100.00%	100.00%	100.00%	100.00%	100.00%		
Yes	39	24	31	185	144	123		
	79.60%	58.50%	59.60%	17.00%	13.60%	11.70%	65.9%	45.0%
No	10	14	15	829	803	824		
	20.40%	34.10%	28.80%	76.20%	76.00%	78.60%	27.8%	76.9%
Don't know/not sure	-	3	6	73	107	99		
	-	7.30%	11.50%	6.70%	10.10%	9.40%	9.4%	8.7%
Decline to answer	-	-	-	1	3	2		
	-	-	-	0.10%	0.30%	0.20%		

Table 12	Autistic			Non-Autistic			Autistic US, AU, UK	Non-Autistic US, AU, UK
BA. Which of the following health problems do you experience? Base: Respondents who experienced below health problems from being in a room after it has been cleaned with scented products								
	US	AU	UK	US	AU	UK		
Total	39	24	31	185	144	123		
	100.00%	100.00%	100.00%	100.00%	100.00%	100.00%		
Migraine headaches	12	8	12	63	30	29		
	30.80%	33.30%	38.70%	34.10%	20.80%	23.60%	34.3%	26.2%
Asthma attacks	7	7	9	39	20	24		
	17.90%	29.20%	29.00%	21.10%	13.90%	19.50%	25.4%	18.2%
Neurological problems (e.g., dizziness, seizures,	14	6	8	33	11	10		
head pain, fainting, loss of coordination)	35.90%	25.00%	25.80%	17.80%	7.60%	8.10%	28.9%	11.2%
Respiratory problems (e.g., difficulty breathing,	11	9	10	98	73	56		
coughing, shortness of breath)	28.20%	37.50%	32.30%	53.00%	50.70%	45.50%	32.7%	49.7%
Skin problems (e.g., rashes, hives, red skin,	12	9	10	33	22	26		
tingling skin, dermatitis)	30.80%	37.50%	32.30%	17.80%	15.30%	21.10%	33.5%	18.1%
Cognitive problems (e.g., difficulties thinking,	12	9	7	19	11	9		
concentrating, or remembering)	30.80%	37.50%	22.60%	10.30%	7.60%	7.30%	30.3%	8.4%
Mucosal symptoms (e.g., watery or red eyes, nasal	11	9	8	72	57	33		
congestion, sneezing)	28.20%	37.50%	25.80%	38.90%	39.60%	26.80%	30.5%	35.1%
Immune system problems (e.g., swollen lymph	13	12	2	10	6	7		
glands, fever, fatigue)	33.30%	50.00%	6.50%	5.40%	4.20%	5.70%	29.9%	5.1%
Gastrointestinal problems (e.g., nausea, bloating,	7	7	5	25	9	6		
cramping, diarrhea)	17.90%	29.20%	16.10%	13.50%	6.30%	4.90%	21.1%	8.2%
Cardiovascular problems (e.g., fast or irregular	12	6	9	14	8	2		
heartbeat, jitteriness, chest discomfort)	30.80%	25.00%	29.00%	7.60%	5.60%	1.60%	28.3%	4.9%
Musculoskeletal problems (e.g., muscle or joint	12	8	6	11	3	1		
pain, cramps, weakness)	30.80%	33.30%	19.40%	5.90%	2.10%	0.80%	27.8%	2.9%
Other	1	-	-	3	7	9		
	2.60%	-	-	1.60%	4.90%	7.30%	2.6%	4.6%

Table 13	Autistic			Non-Autistic			Autistic US, AU, UK	Non-Autistic US, AU, UK
	US	AU	UK	US	AU	UK		
**Total**	49	41	52	1088	1057	1048		
	100.00%	100.00%	100.00%	100.00%	100.00%	100.00%		
**Yes**	32	24	30	236	189	121		
	65.30%	58.50%	57.70%	21.70%	17.90%	11.50%	60.5%	17.0%
**No**	15	14	15	784	779	857		
	30.60%	34.10%	28.80%	72.10%	73.70%	81.80%	31.2%	75.9%
**Don't know/not sure**	2	3	7	66	87	69		
	4.10%	7.30%	13.50%	6.10%	8.20%	6.60%	8.3%	7.0%
**Decline to answer**	-	-	-	2	2	1		
	-	-	-	0.20%	0.20%	0.10%		

Q5. Do you experience any health problems from being near someone who is wearing a fragranced product?

Table 14

BA. Which of the following health problems do you experience?
Base: Respondents who experienced below health problems from being near someone who is wearing a fragranced product

	Autistic			Non-Autistic			Autistic US, AU, UK	Non-Autistic US, AU, UK
	US	AU	UK	US	AU	UK		
Total	32	24	30	236	189	121		
	100.00%	100.00%	100.00%	100.00%	100.00%	100.00%		
Migraine headaches	8	8	8	88	46	29		
	25.00%	33.30%	26.70%	37.30%	24.30%	24.00%	28.3%	28.5%
Asthma attacks	7	8	6	37	29	19		
	21.90%	33.30%	20.00%	15.70%	15.30%	15.70%	25.1%	15.6%
Neurological problems (e.g., dizziness, seizures,	10	4	8	31	22	5		
head pain, fainting, loss of coordination)	31.30%	16.70%	26.70%	13.10%	11.60%	4.10%	24.9%	9.6%
Respiratory problems (e.g., difficulty breathing,	12	6	7	106	85	39		
coughing, shortness of breath)	37.50%	25.00%	23.30%	44.90%	45.00%	32.20%	28.6%	40.7%
Skin problems (e.g., rashes, hives, red skin,	13	7	4	26	12	22		
tingling skin, dermatitis)	40.60%	29.20%	13.30%	11.00%	6.30%	18.20%	27.7%	11.8%
Cognitive problems (e.g., difficulties thinking,	14	5	7	16	12	5		
concentrating, or remembering)	43.80%	20.80%	23.30%	6.80%	6.30%	4.10%	29.3%	5.7%
Mucosal symptoms (e.g., watery or red eyes, nasal	8	10	6	90	77	45		
congestion, sneezing)	25.00%	41.70%	20.00%	38.10%	40.70%	37.20%	28.9%	38.7%
Immune system problems (e.g., swollen lymph	9	9	4	10	5	1		
glands, fever, fatigue)	28.10%	37.50%	13.30%	4.20%	2.60%	0.80%	26.3%	2.5%
Gastrointestinal problems (e.g., nausea, bloating,	9	5	6	22	11	6		
cramping, diarrhea)	28.10%	20.80%	20.00%	9.30%	5.80%	5.00%	23.0%	6.7%
Cardiovascular problems (e.g., fast or irregular	9	4	9	11	9	4		
heartbeat, jitteriness, chest discomfort)	28.10%	16.70%	30.00%	4.70%	4.80%	3.30%	24.9%	4.3%
Musculoskeletal problems (e.g., muscle or joint	12	10	7	5	3	4		
pain, cramps, weakness)	37.50%	41.70%	23.30%	2.10%	1.60%	3.30%	34.2%	2.3%
Other	1	-	-	6	9	6		
	3.10%	-	-	2.50%	4.80%	5.00%	3.1%	4.1%

Table 15

Q6. In general, do you experience any health problems from exposure to any type of fragranced product?

	Autistic			Non-Autistic			Autistic US, AU, UK	Non-Autistic US, AU, UK
	US	AU	UK	US	AU	UK		
**Total**	49	41	52	1088	1057	1048		
	100.00%	100.00%	100.00%	100.00%	100.00%	100.00%		
**Yes**	36	30	24	217	193	129		
	73.50%	73.20%	46.20%	19.90%	18.30%	12.30%	64.3%	16.8%
**No**	12	10	19	784	768	811		
	24.50%	24.40%	36.50%	72.10%	72.70%	77.40%	28.5%	74.1%
**Don't know/not sure**	1	1	9	86	95	107		
	2.00%	2.40%	17.30%	7.90%	9.00%	10.20%	7.2%	9.0%
**Decline to answer**	-	-	-	1	1	1		
	-	-	-	0.10%	0.10%	0.10%		

Table 16	Autistic			Non-Autistic			Autistic US, AU, UK	Non-Autistic US, AU, UK
BA. Which of the following health problems do you experience?<br>Base: Respondents who experienced below health problems from exposure to any type of fragranced product								
	US	AU	UK	US	AU	UK		
Total	36	30	24	217	193	129		
	100.00%	100.00%	100.00%	100.00%	100.00%	100.00%		
Migraine headaches	9	8	7	89	43	26		
	25.00%	26.70%	29.20%	41.00%	22.30%	20.20%	27.0%	27.8%
Asthma attacks	8	9	5	47	31	25		
	22.20%	30.00%	20.80%	21.70%	16.10%	19.40%	24.3%	19.1%
Neurological problems (e.g., dizziness, seizures,	17	5	5	25	17	10		
head pain, fainting, loss of coordination)	47.20%	16.70%	20.80%	11.50%	8.80%	7.80%	28.2%	9.4%
Respiratory problems (e.g., difficulty breathing,	13	11	7	106	99	44		
coughing, shortness of breath)	36.10%	36.70%	29.20%	48.80%	51.30%	34.10%	34.0%	44.7%
Skin problems (e.g., rashes, hives, red skin,	15	11	4	43	43	41		
tingling skin, dermatitis)	41.70%	36.70%	16.70%	19.80%	22.30%	31.80%	31.7%	24.6%
Cognitive problems (e.g., difficulties thinking,	13	7	4	18	12	9		
concentrating, or remembering)	36.10%	23.30%	16.70%	8.30%	6.20%	7.00%	25.4%	7.2%
Mucosal symptoms (e.g., watery or red eyes, nasal	12	10	5	90	73	43		
congestion, sneezing)	33.30%	33.30%	20.80%	41.50%	37.80%	33.30%	29.1%	37.5%
Immune system problems (e.g., swollen lymph	11	7	4	13	6	7		
glands, fever, fatigue)	30.60%	23.30%	16.70%	6.00%	3.10%	5.40%	23.5%	4.8%
Gastrointestinal problems (e.g., nausea, bloating,	7	3	7	26	11	4		
cramping, diarrhea)	19.40%	10.00%	29.20%	12.00%	5.70%	3.10%	19.5%	6.9%
Cardiovascular problems (e.g., fast or irregular	10	5	7	8	8	6		
heartbeat, jitteriness, chest discomfort)	27.80%	16.70%	29.20%	3.70%	4.10%	4.70%	24.6%	4.2%
Musculoskeletal problems (e.g., muscle or joint	13	9	7	7	2	3		
pain, cramps, weakness)	36.10%	30.00%	29.20%	3.20%	1.00%	2.30%	31.8%	2.2%
Other	1	-	-	2	8	10		
	2.80%	-	-	0.90%	4.10%	7.80%	2.8%	4.3%

Table 17	Autistic			Non-Autistic			Autistic US, AU, UK	Non-Autistic US, AU, UK

Disability definition question:

US: Do any of these health problems substantially limit one or more major life activities, such as seeing, hearing, eating, sleeping, walking, standing, lifting, bending, speaking, breathing, learning, reading, concentrating, thinking, communicating, or working, for you personally?

AU: Do any of these health problems mean a total or partial loss of bodily or mental functions, for you personally?

UK: Do any of these health problems cause a substantial, likely to recur, and adverse effect on your ability to carry out normal day-to-day activities?

	US	AU	UK	US	AU	UK		
**Total**	41	34	44	353	328	262		
	100.00%	100.00%	100.00%	100.0%	100.00%	100.00%		
**Yes**	35	28	24	160	34	54		
	85.40%	82.40%	54.50%	45.3%	10.40%	20.60%	74.1%	25.4%
**No**	5	6	14	170	263	181		
	12.20%	17.60%	31.80%	48.2%	80.20%	69.10%	20.5%	65.8%
**Don't know/not sure**	1	-	6	21	31	27		
	2.40%	-	13.60%	5.9%	9.50%	10.30%	8.0%	8.6%
**Decline to answer**	-	-	-	2	-	-		
	-	-	-	0.6%	-	-		

Table 18	Autistic			Non-Autistic			Autistic US, AU, UK	Non-Autistic US, AU, UK
Has a doctor or health care professional ever told you that you have autism or autism spectrum disorder?	US	AU	UK	US	AU	UK		
**Total**	49	41	52	1088	1057	1048		
	100.00%	100.00%	100.00%	100.00%	100.00%	100.00%		
**Yes - autism**	25	24	27	-	-	-		
	51.00%	58.50%	51.90%	-	-	-	53.8%	
**Yes - autism spectrum disorder**	26	26	29	-	-	-		
	53.10%	63.40%	55.80%	-	-	-	57.4%	
**No**	-	-	-	1073	1025	1021		
	-	-	-	98.60%	97.00%	97.40%		97.7%
**Don't know/not sure**	-	-	-	13	30	26		
	-	-	-	1.20%	2.80%	2.50%		2.2%
**Decline to answer**	-	-	-	2	2	1		
	-	-	-	0.20%	0.20%	0.10%		0.2%
Percentages below relative to general population:	1137	1098	1100	1137	1098	1100		
autism or autism spectrum disorder	49	41	52				4.3%	
	4.3%	3.7%	4.7%					
autism	25	24	27				2.3%	
	2.2%	2.2%	2.5%					
autism spectrum disorder	26	26	29				2.4%	
	2.3%	2.4%	2.6%					
not autistic				1088	1057	1048		95.7%
				95.7%	96.3%	95.3%		
not autism or autism spectrum disorder				1073	1025	1021		93.5%
				94.4%	93.4%	92.8%		
don't know/not sure				13	30	26		2.1%
				1.1%	2.7%	2.4%		
decline to answer				2	2	1		0.1%
				0.2%	0.2%	0.1%		

Table 19	Autistic			Non-Autistic			Autistic US, AU, UK	Non-Autistic US, AU, UK
Have you ever been unable or reluctant to use the restrooms in a public place, because of the presence of an air freshener, deodorizer, or scented product?								
	US	AU	UK	US	AU	UK		
Total	49	41	52	1088	1057	1048		
	100.00%	100.00%	100.00%	100.00%	100.00%	100.00%		
Yes	36	25	27	163	102	106		
	73.50%	61.00%	51.90%	15.00%	9.60%	10.10%	62.1%	11.6%
No	12	13	20	885	907	871		
	24.50%	31.70%	38.50%	81.30%	85.80%	83.10%	31.6%	83.4%
Neutral/not sure	1	3	4	39	46	68		
	2.00%	7.30%	7.70%	3.60%	4.40%	6.50%	5.7%	4.8%
Decline to answer	-	-	1	1	2	3		
	-	-	1.90%	0.10%	0.20%	0.30%		

Table 20	Autistic			Non-Autistic			Autistic US, AU, UK	Non-Autistic US, AU, UK
If you enter a business, and you smell air fresheners or some fragranced product, do you want to leave as quickly as possible?	US	AU	UK	US	AU	UK		
Total	49	41	52	1088	1057	1048		
	100.00%	100.00%	100.00%	100.00%	100.00%	100.00%		
Yes	31	25	27	198	158	117		
	63.30%	61.00%	51.90%	18.20%	14.90%	11.20%	58.7%	14.8%
No	14	14	15	773	759	812		
	28.60%	34.10%	28.80%	71.00%	71.80%	77.50%	30.5%	73.4%
Neutral/not sure	4	2	10	116	139	115		
	8.20%	4.90%	19.20%	10.70%	13.20%	11.00%	10.8%	11.6%
Decline to answer	-	-	-	1	1	4		
	-	-	-	0.10%	0.10%	0.40%		

Table 21	Autistic			Non-Autistic			Autistic US, AU, UK	Non-Autistic US, AU, UK
Have you ever been unable or reluctant to wash your hands with soap in a public place, because you know or suspect that the soap is fragranced?								
	US	AU	UK	US	AU	UK		
Total	49	41	52	1088	1057	1048		
	100.00%	100.00%	100.00%	100.00%	100.00%	100.00%		
Yes	29	28	27	131	85	86		
	59.20%	68.30%	51.90%	12.00%	8.00%	8.20%	59.8%	9.4%
No	16	13	20	908	898	879		
	32.70%	31.70%	38.50%	83.50%	85.00%	83.90%	34.3%	84.1%
Neutral/not sure	3	-	4	47	72	78		
	6.10%	-	7.70%	4.30%	6.80%	7.40%	6.9%	6.2%
Decline to answer	1	-	1	2	2	5		
	2.00%	-	1.90%	0.20%	0.20%	0.50%	2.0%	0.3%

Table 22	Autistic			Non-Autistic			Autistic US, AU, UK	Non-Autistic US, AU, UK
Have you ever been prevented from going to some place because you would be exposed to a fragrance product that would make you sick?								
	US	AU	UK	US	AU	UK		
Total	49	41	52	1088	1057	1048		
	100.00%	100.00%	100.00%	100.00%	100.00%	100.00%		
Yes	37	29	28	221	136	120		
	75.50%	70.70%	53.80%	20.30%	12.90%	11.50%	66.7%	14.9%
No	11	9	20	808	823	853		
	22.40%	22.00%	38.50%	74.30%	77.90%	81.40%	27.6%	77.9%
Don't know/not sure	-	3	3	58	96	73		
	-	7.30%	5.80%	5.30%	9.10%	7.00%	6.6%	7.1%
Decline to answer	1	-	1	1	2	2		
	2.00%	-	1.90%	0.10%	0.20%	0.20%	2.0%	0.2%
SUM	49	41	52					
	100.00%	100.00%	100.00%					

Table 23	Autistic			Non-Autistic			Autistic US, AU, UK	Non-Autistic US, AU, UK
**Has any exposure to fragranced products in your work environment caused you to become sick, lose work days, or lose a job?**								
	US	AU	UK	US	AU	UK		
**Total**	49	41	52	1088	1057	1048		
	100.00%	100.00%	100.00%	100.00%	100.00%	100.00%		
**Yes**	35	28	20	137	57	49		
	71.40%	68.30%	38.50%	12.60%	5.40%	4.70%	59.4%	7.6%
**No**	12	11	23	898	918	930		
	24.50%	26.80%	44.20%	82.50%	86.80%	88.70%	31.8%	86.0%
**Don't know/not sure**	2	2	8	52	79	66		
	4.10%	4.90%	15.40%	4.80%	7.50%	6.30%	8.1%	6.2%
**Decline to answer**	-	-	1	1	3	3		
	-	-	1.90%	0.10%	0.30%	0.30%		

Table 24	Autistic			Non-Autistic			Autistic US, AU, UK	Non-Autistic US, AU, UK
Would you be supportive of a fragrance-free policy in the workplace?								
	US	AU	UK	US	AU	UK		
Total	49	41	52	1088	1057	1048		
	100.00%	100.00%	100.00%	100.00%	100.00%	100.00%		
Yes	28	32	32	576	438	460		
	57.10%	78.00%	61.50%	52.90%	41.40%	43.90%	65.5%	46.1%
No	13	6	16	211	238	240		
	26.50%	14.60%	30.80%	19.40%	22.50%	22.90%	24.0%	21.6%
Neutral/not sure	8	3	4	296	378	340		
	16.30%	7.30%	7.70%	27.20%	35.80%	32.40%	10.4%	31.8%
Decline to answer	-	-	-	5	3	8		
	-	-	-	0.50%	0.30%	0.80%		

Table 25	Autistic			Non-Autistic			Autistic US, AU, UK	Non-Autistic US, AU, UK
Would you prefer that health care facilities and health care professionals be fragrance-free?								
	US	AU	UK	US	AU	UK		
Total	49	41	52	1088	1057	1048		
	100.00%	100.00%	100.00%	100.00%	100.00%	100.00%		
Yes	39	34	36	584	440	440		
	79.60%	82.90%	69.20%	53.70%	41.60%	42.00%	77.2%	45.8%
No	5	5	14	250	272	280		
	10.20%	12.20%	26.90%	23.00%	25.70%	26.70%	16.4%	25.1%
Neutral/not sure	4	2	2	250	340	322		
	8.20%	4.90%	3.80%	23.00%	32.20%	30.70%	5.6%	28.6%
Decline to answer	1	-	-	4	5	6		
	2.00%	-	-	0.40%	0.50%	0.60%	2.0%	0.5%

Table 26	Autistic			Non-Autistic			Autistic US, AU, UK	Non-Autistic US, AU, UK
People Who Answer "Yes" To One Or More Of These Questions: Q2/Q3/Q4/Q5/Q6 (fragrance sensitive group).								
	US	AU	UK	US	AU	UK		
Total	49	41	52	1088	1057	1048	142	3193
	100.00%	100.00%	100.00%	100.00%	100.00%	100.00%		
Yes	41	34	44	353	328	262	119	943
	83.70%	82.90%	84.60%	32.40%	31.00%	25.00%	83.7%	29.5%

Table 27	Autistic			Non-Autistic			Autistic US, AU, UK	Non-Autistic US, AU, UK

People who answer "Yes" to each type of health problem for each of these questions Q2/Q3/Q4/Q5/Q6.

	Autistic			Non-Autistic			Autistic	Non-Autistic
	US	AU	UK	US	AU	UK	US, AU, UK	US, AU, UK
Total	49	41	52	1088	1057	1048		
	100.00%	100.00%	100.00%	100.00%	100.00%	100.00%		
Migraine headaches	22	17	22	157	93	70		
	44.90%	41.50%	42.30%	14.40%	8.80%	6.70%	42.9%	10.0%
Asthma attacks	14	19	17	77	64	58		
	28.60%	46.30%	32.70%	7.10%	6.10%	5.50%	35.9%	6.2%
Neurological problems (e.g., dizziness, seizures,	20	12	17	62	37	24		
head pain, fainting, loss of coordination)	40.80%	29.30%	32.70%	5.70%	3.50%	2.30%	34.3%	3.8%
Respiratory problems (e.g., difficulty breathing,	23	20	20	188	163	108		
coughing, shortness of breath)	46.90%	48.80%	38.50%	17.30%	15.40%	10.30%	44.7%	14.3%
Skin problems (e.g., rashes, hives, red skin,	19	18	19	102	86	89		
tingling skin, dermatitis)	38.80%	43.90%	36.50%	9.40%	8.10%	8.50%	39.7%	8.7%
Cognitive problems (e.g., difficulties thinking,	20	13	13	46	32	18		
concentrating, or remembering)	40.80%	31.70%	25.00%	4.20%	3.00%	1.70%	32.5%	3.0%
Mucosal symptoms (e.g., watery or red eyes, nasal	21	20	18	163	134	83		
congestion, sneezing)	42.90%	48.80%	34.60%	15.00%	12.70%	7.90%	42.1%	11.9%
Immune system problems (e.g., swollen lymph	18	18	7	27	18	14		
glands, fever, fatigue)	36.70%	43.90%	13.50%	2.50%	1.70%	1.30%	31.4%	1.8%
Gastrointestinal problems (e.g., nausea, bloating,	15	10	17	48	26	16		
cramping, diarrhea)	30.60%	24.40%	32.70%	4.40%	2.50%	1.50%	29.2%	2.8%
Cardiovascular problems (e.g., fast or irregular	18	13	18	32	20	17		
heartbeat, jitteriness, chest discomfort)	36.70%	31.70%	34.60%	2.90%	1.90%	1.60%	34.3%	2.1%
Musculoskeletal problems (e.g., muscle or joint	19	15	14	24	14	8		
pain, cramps, weakness)	38.80%	36.60%	26.90%	2.20%	1.30%	0.80%	34.1%	1.4%
Other	1	-	1	18	21	22		
	2.00%	-	1.90%	1.70%	2.00%	2.10%	2.0%	1.9%

Table 28

Demographics.

Base: Autistic, Non-Austistic

	Autistic			Non-Autistic			Autistic US, AU, UK	Non-Autistic US, AU, UK
	US	AU	UK	US	AU	UK		
**Total**	49	41	52	1088	1057	1048		
	100.00%	100.00%	100.00%	100.00%	100.00%	100.00%		
**Male/Female**								
**All Males**	33	24	34	492	519	516		
	67.30%	58.50%	65.40%	45.20%	49.10%	49.20%	63.7%	47.8%
**All Females**	16	17	18	596	538	532		
	32.70%	41.50%	34.60%	54.80%	50.90%	50.80%	36.3%	52.2%
**Gender vs Age**								
**Male 18-24**	6	5	6	41	65	77		
	12.20%	12.20%	11.50%	3.80%	6.10%	7.30%	12.0%	5.7%
**Male 25-34**	13	7	15	117	102	87		
	26.50%	17.10%	28.80%	10.80%	9.60%	8.30%	24.1%	9.6%
**Male 35-44**	12	6	8	124	113	99		
	24.50%	14.60%	15.40%	11.40%	10.70%	9.40%	18.2%	10.5%
**Male 45-54**	2	5	4	106	121	131		
	4.10%	12.20%	7.70%	9.70%	11.40%	12.50%	8.0%	11.2%
**Male 55-65**	-	1	1	104	118	122		
	-	2.40%	1.90%	9.60%	11.20%	11.60%	2.2%	10.8%
**Female 18-24**	3	2	4	75	84	77		
	6.10%	4.90%	7.70%	6.90%	7.90%	7.30%	6.2%	7.4%
**Female 25-34**	3	7	10	132	123	119		
	6.10%	17.10%	19.20%	12.10%	11.60%	11.40%	14.1%	11.7%
**Female 35-44**	8	4	3	147	133	132		
	16.30%	9.80%	5.80%	13.50%	12.60%	12.60%	10.6%	12.9%
**Female 45-54**	1	4	1	143	111	107		
	2.00%	9.80%	1.90%	13.10%	10.50%	10.20%	4.6%	11.3%
**Female 55-65**	1	-	-	99	87	97		
	2.00%	-	-	9.10%	8.20%	9.30%	2.0%	8.9%

Table 29

Demographics.

Base: General Population

	US	AU	UK				US, AU, UK
**Total**	1137	1098	1100				
	100.00%	100.00%	100.00%				
**Male/Female**							
**All Males**	525	543	550				
	46.20%	49.50%	50.00%				48.6%
**All Females**	612	555	550				
	53.80%	50.50%	50.00%				51.4%
**Gender vs Age**							
**Male 18-24**	47	70	83				
	4.10%	6.40%	7.50%				6.0%
**Male 25-34**	130	109	102				
	11.40%	9.90%	9.30%				10.2%
**Male 35-44**	136	119	107				
	12.00%	10.80%	9.70%				10.8%
**Male 45-54**	108	126	135				
	9.50%	11.50%	12.30%				11.1%
**Male 55-65**	104	119	123				
	9.10%	10.80%	11.20%				10.4%
**Female 18-24**	78	86	81				
	6.90%	7.80%	7.40%				7.4%
**Female 25-34**	135	130	129				
	11.90%	11.80%	11.70%				11.8%
**Female 35-44**	155	137	135				
	13.60%	12.50%	12.30%				12.8%
**Female 45-54**	144	115	108				
	12.70%	10.50%	9.80%				11.0%
**Female 55-65**	100	87	97				
	8.80%	7.90%	8.80%				8.5%

Table 30	Autistic			Non-Autistic			Autistic US, AU, UK	Non-Autistic US, AU, UK
	US	AU	UK	US	AU	UK		
**Total**	41	34	44	353	328	262	119	943
	100.00%	100.00%	100.00%	100.00%	100.00%	100.00%		
**Male/Female**								
**All Males**	28	21	28	145	123	113		
	68.30%	61.80%	63.60%	41.10%	37.50%	43.10%	64.6%	40.6%
**All Females**	13	13	16	208	205	149		
	31.70%	38.20%	36.40%	58.90%	62.50%	56.90%	35.4%	59.4%
**Gender vs Age**								
**Male 18-24**	4	3	6	10	14	14		
	9.80%	8.80%	13.60%	2.80%	4.30%	5.30%	10.7%	4.1%
**Male 25-34**	11	7	14	36	35	25		
	26.80%	20.60%	31.80%	10.20%	10.70%	9.50%	26.4%	10.1%
**Male 35-44**	11	6	7	44	22	23		
	26.80%	17.60%	15.90%	12.50%	6.70%	8.80%	20.1%	9.3%
**Male 45-54**	2	5	-	30	29	27		
	4.90%	14.70%	-	8.50%	8.80%	10.30%	9.8%	9.2%
**Male 55-65**	-	-	1	25	23	24		
	-	-	2.30%	7.10%	7.00%	9.20%	2.3%	7.8%
**Female 18-24**	3	2	3	17	25	19		
	7.30%	5.90%	6.80%	4.80%	7.60%	7.30%	6.7%	6.6%
**Female 25-34**	2	5	9	48	38	32		
	4.90%	14.70%	20.50%	13.60%	11.60%	12.20%	13.4%	12.5%
**Female 35-44**	7	2	3	54	53	40		
	17.10%	5.90%	6.80%	15.30%	16.20%	15.30%	9.9%	15.6%
**Female 45-54**	1	4	1	52	45	32		
	2.40%	11.80%	2.30%	14.70%	13.70%	12.20%	5.5%	13.5%
**Female 55-65**	-	-	-	37	44	26		
	-	-	-	10.50%	13.40%	9.90%		11.3%

Demographics.

Base: Autistic, Non-Austistic: Fragrance Sensitive
